# Patterns of 25-Hydroxyvitamin D3, Calcium Status, and Anemia in the Saudi Population: A Cross-Sectional Study

**DOI:** 10.3390/life12122119

**Published:** 2022-12-15

**Authors:** Mohammad A. Alfhili, Ahmed M. Basudan, Mohammed Alfaifi, Zuhier A. Awan, Mohammed R. Algethami, Jawaher Alsughayyir

**Affiliations:** 1 Department of Clinical Laboratory Sciences, College of Applied Medical Sciences, King Saud University, Riyadh 12372, Saudi Arabia; 2 Department of Clinical Laboratory Sciences, College of Applied Medical Sciences, King Khalid University, Abha 61421, Saudi Arabia; 3 Department of Clinical Biochemistry, Faculty of Medicine, King Abdulaziz University, Jeddah 21589, Saudi Arabia; 4 Al-Borg Medical Laboratories, Department of Clinical Pathology, Jeddah 23437, Saudi Arabia; 5 Preventive Medicine and Public Health, Ministry of Health, Jeddah 23325, Saudi Arabia

**Keywords:** vitamin D, calcium, anemia, biomarker, Saudi Arabia

## Abstract

Background: Emerging evidence suggests an intricate relationship between vitamin D, Ca^2+^, and inflammation-driven anemia. We, thus, investigated the patterns of serum 25(OH)D_3_, Ca^2+^, ferritin, and iron in healthy and anemic members of the Saudi population. Methods: A population-based, retrospective, cross-sectional study was designed to analyze data for 14,229 subjects, aged 3–110 years, obtained from Al-Borg Medical Laboratories, over a six-year period (2014–2020). Gender and age differences were analyzed for 25(OH)D_3_, Ca^2+^, hemoglobin, ferritin, and iron. Results: Vitamin D deficiency was extremely prevalent (98.47%) irrespective of age or gender, despite an increasing trend with age, in clear contrast to serum Ca^2+^. Ferritin was significantly lower in young adult and adult females, compared to elderly females, whereas iron was significantly reduced in females; in particular, adult females compared to young adults or elderly adults. Only anemic adult males had significantly lower 25(OH)D_3_, while Ca^2+^ was consistently significantly diminished in anemics of all age groups, independent of gender. Notably, hypocalcemic subjects were 2.36 times more likely to be anemic. Moreover, ferritin, but not iron, was significantly diminished in anemics, which was only evident in young adults and adults. However, both ferritin and iron showed positive correlation with hematocrit, hemoglobin, MCH, MCHC, and MCV. Conclusions: Despite being significantly lower in anemics, 25(OH)D_3_ is not particularly associated with anemia, while hypocalcemia is associated with an increased risk for anemia. Assessment of vitamin D and Ca^2+^ status may be valuable in the clinical management of anemia in the Saudi population.

## 1. Introduction

Anemia is an extremely common and potentially life-threatening condition of public health concern. A multifactorial condition, anemia is characterized by diminished ability of red blood cells to transport oxygen. This may arise due to aberrant or inadequate RBC production, blood loss, or premature RBC death [1]. Hereditary, nutritional, inflammatory, and drug-related causes have been established, of which, iron deficiency is the most common, followed by chronic disease. Instigated and sustained by inflammatory mediators, anemia of chronic disease is a complication of diabetes, infections, malignancy, chronic kidney disease, and autoimmunity, among others [2].

Vitamin D_3_ is a cholesterol derivative, cholecalciferol endogenously synthesized following exposure to sunlight. In the skin, 7-dehydro-cholesterol is metabolized to vitamin D_3_ which is converted to 25-hydroxycholecalciferol (25(OH)D_3_) in the liver, which is finally hydroxylated in the kidneys to the active 1,25-dihydroxycholecalciferol (1,25-[OH]_2_-D_3_). Vitamin D elevates serum Ca^2+^ as it promotes its intestinal absorption and augments bone resorption induced by parathyroid hormone (PTH). Serum level of 25(OH)D_3_ reflects body stores and, hence, is routinely used to assess deficiency. Previous reports indicated that the prevalence of vitamin D deficiency (VDD) in the Saudi population is alarmingly high, ranging from 30% to 100% [3]. While the exact etiology behind this high prevalence remains elusive, contributing factors include genetic predisposition, chronic disease, dietary habits, and sedentary lifestyle [4].

Ca^2+^ is the most abundant mineral in the human body, most of which is present in the bones, complexed with phosphate as hydroxyapatite. Ca^2+^ is essential for muscle contraction, nerve impulses, blood clotting, and for cell motility, differentiation, and death. Serum Ca^2+^ is maintained by two opposing hormones, namely, calcitonin and PTH. Hypocalcemia leads to neuromuscular disturbances manifested as tetany and osteodystrophy, and may arise secondarily to hypoalbuminemia, VDD, acute pancreatitis, and others. Hypercalcemia, a major cause of renal lithiasis, is often due to increased vitamin D, hyperthyroidism, and malignancy [5].

Since Ca^2+^ is in large part regulated by vitamin D, disturbances in these two parameters are closely related. Moreover, emerging evidence points at a potential role of vitamin D in iron homeostasis and anemia. Many reports have demonstrated an association between vitamin D availability and protection from anemia, particularly, anemia that ensues within an inflammatory milieu [6]. Nevertheless, the identity and spectrum of effect modifiers governing this association remain, for the most part, elusive. This report, therefore, aimed to further characterize the influence of age and gender on the interplay among vitamin D, Ca^2+^ status, and anemia in the Saudi population.

## 2. Materials and Methods

### 2.1. Study Design and Population

This cross-sectional study was approved by the Biomedical Ethics Unit of Al-Borg Medical Laboratories (Approval #07/21). Age, gender, and laboratory data for a total of 14,229 subjects were retrieved from Al-Borg Medical Laboratories database and analyzed. Each subject in the database was assigned a unique ID, against which a single reading for all parameters was recorded, so all subjects were considered only once. Subjects with missing information necessary for a particular analysis were excluded. Subjects were stratified, based on gender and age group, into young (0–17 years), young adults (18–39 years), adults (40–64 years), and elderlies (65–110 years) [7,8], as shown in Table 1.

To analyze 25(OH)D_3_ status, cutoff values previously reported for Saudis were used [3]. Based on a reference range of 50–75 nmol/L, sufficiency was defined as ≥75 nmol/L, insufficiency as 50.1–74.9 nmol/L, and deficiency as ≤50 nmol/L. Anemia was defined by a Hb level of ≤12 mg/dL for young females, <11.5 mg/dL for all other age groups of females, and <13.5 mg/dL for males [9,10]. Reference ranges of 8.5–10.5 mg/dL for total Ca^2+^ [11], of 50–170 μg/dL for iron [12], and of 24–336 μg/L (males) and 11–307 μg/L (females) for ferritin were used.

### 2.2. Statistical Analysis

Means ± standard error of the mean (SEM) was compared among genders and age groups. Two groups were analyzed by Student’s *t*-test while one-way ANOVA with Tukey’s honest significance test was employed to compare the means among three or more groups. Associations of continuous variables were assessed by Pearson correlation and simple linear regression analysis, and absolute risk, prevalence ratio (PR), and odds ratio (OR) were calculated as permitted by the natural sampling design of this study. All analyses were performed by GraphPad Prism v9.2.0 (GraphPad Software, Inc., San Diego, CA, USA), and a *p* value of <0.05 was considered statistically significant.

## 3. Results

### 3.1. Effect of Gender and Age on 25(OH)D_3_ and Ca^2+^ Levels

**Figure 1:**
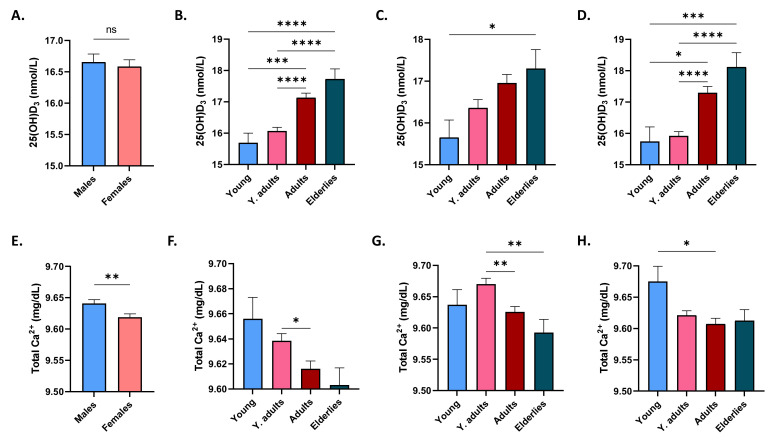
Effect of gender and age on 25(OH)D_3_ and Ca^2+^ levels. Mean ± SEM of serum 25(OH)D_3_ in males and females (**A**), in age groups of both genders (**B**), of males (**C**), and of females (**D**). Mean ± SEM of serum Ca^2+^ in males and females (**E**), in age groups of both genders (**F**), of males (**G**), and of females (**H**). * (*p* < 0.05), ** (*p* < 0.01), *** (*p* < 0.001), and **** (*p* < 0.0001) indicate significant difference, while ns indicates no significance.

The mean 25(OH)D_3_ level in males and females was not significantly different (16.6 ± 0.12 nmol/L and 16.5 ± 0.11 nmol/L, respectively) as shown in Figure 1A. When age groups were analyzed in both genders, significant differences in 25(OH)D_3_ were found between the young (15.70 ± 0.30 nmol/L) and adults (17.14 ± 0.14 nmol/L) and the young and elderlies (17.73 ± 0.32 nmol/L). Young adults also had significantly lower 25(OH)D_3_ (16.07 ± 0.11 nmol/L) than adults and elderlies (Figure 1B). However, when only males were considered (Figure 1C), only the significant increase between the young and elderlies persisted (15.65 ± 0.41 nmol/L to 17.30 ± 0.45 nmol/L). In females, the same pattern observed when both genders were considered was restored (Figure 1D).

Figure 1E shows that total Ca^2+^ was significantly lower in females than in males (9.64 ± 0.01 mg/dL to 9.61 ± 0.01 mg/dL). A progressive decrease in total Ca^2+^ levels was noted with age (Figure 1F), albeit only showing statistical significance between young adults and adults (9.63 ± 0.01 mg/dL to 9.61 ± 0.01 mg/dL). This was also true in males in addition to a significant decrease between young adults and elderlies (9.67 ± 0.01 mg/dL to 9.59 ± 0.02 mg/dL), as depicted in Figure 1G. The only two groups significantly different from each other in females were the young and adults (9.67 ± 0.02 mg/dL to 9.61 ± 0.02 mg/dL), as seen in Figure 1H.

### 3.2. Effect of Gender and Age on Ferritin and Iron Levels

**Figure 2:**
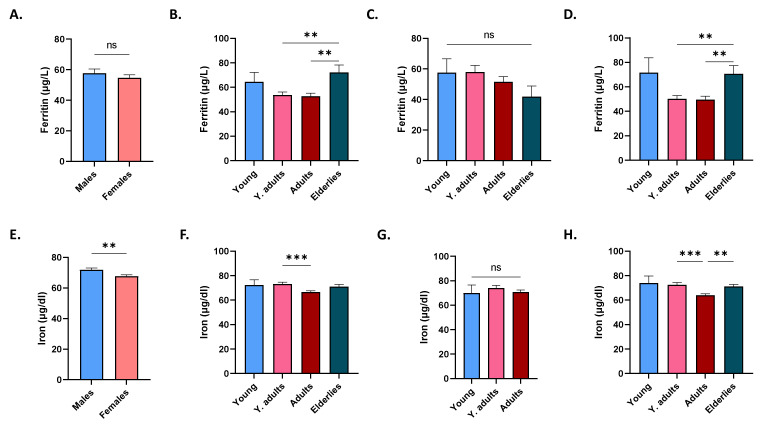
Effect of gender and age on ferritin and iron levels. Mean ± SEM of serum ferritin in males and females (**A**), in age groups of both genders (**B**), of males (**C**), and of females (**D**). Mean ± SEM of serum iron in males and females (**E**), in age groups of both genders (**F**), of males (**G**), and of females (**H**). ** (*p* < 0.01) and *** (*p* < 0.001) indicate significant difference, while ns indicates no significance.

Ferritin levels did not significantly differ between genders (Figure 2A) but were significantly higher in elderlies compared to young adults and adults (Figure 2B), which was preserved only in females (Figure 2C,D). Conversely, iron was significantly lower in females (Figure 2E), in adults compared to young adults (Figure 2F), and in female adults, comparing young adults and elderlies (Figure 2G,H).

### 3.3. Distribution of Hb Level in Study Subjects

**Figure 3:**
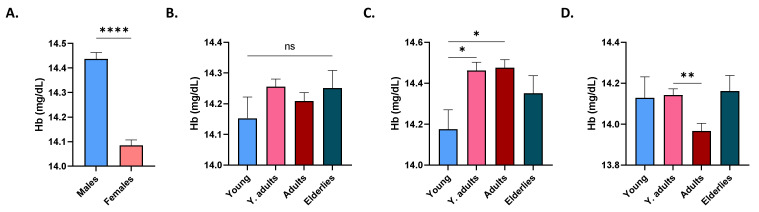
Distribution of Hb level in study subjects. Mean ± SEM of Hb levels in males and females (**A**), in age groups of both genders (**B**), of males (**C**), and of females (**D**). * (*p* < 0.05), ** (*p* < 0.01), and **** (*p* < 0.0001) indicate significant difference, while ns indicates no significance.

As shown in Figure 3A, the mean Hb level was significantly lower in females (14.08 ± 0.02 mg/dL) compared to males (14.44 ± 0.02 mg/dL). No significant difference was detected among age groups when both genders were considered (Figure 3B), but, upon exclusion of females, (Figure 3C), the young had a significantly lower Hb level (14.18 ± 0.09 mg/dL) than young adults (14.46 ± 0.04 mg/dL) and adults (14.48 ± 0.03 mg/dL). In females, adults (13.97 ± 0.03 mg/dL) had significantly lower values than young adults (14.14 ± 0.03 mg/dL; Figure 3D).

### 3.4. Patterns of Serum 25(OH)D_3_ in Anemic Subjects

**Figure 4:**
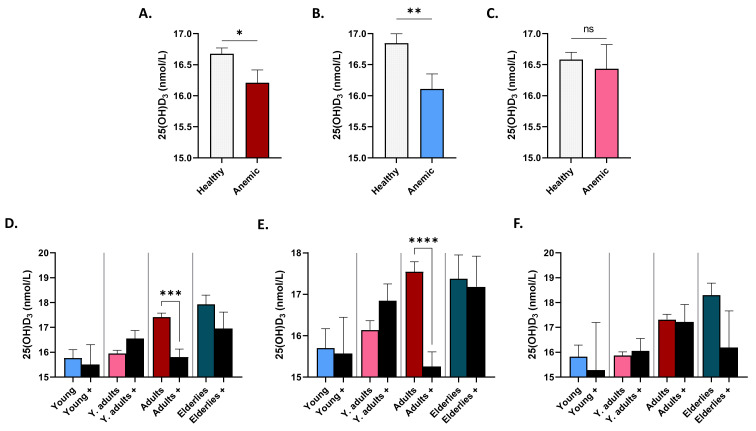
25(OH)D_3_ levels in healthy and anemic subjects. Mean ± SEM of serum 25(OH)D_3_ in healthy and anemics (+) of both genders (**A**), of males (**B**), of females (**C**), in age groups of both genders (**D**), of males (**E**), and of females (**F**). * (*p* < 0.05), ** (*p* < 0.01), *** (*p* < 0.001), and **** (*p* < 0.0001) indicate significant difference, while ns indicates no significance.

As depicted in Figure 4A, mean serum levels of 25(OH)D_3_ were significantly lower in anemic subjects (16.21 ± 0.20 nmol/L) compared to non-anemic controls (16.68 ± 0.09 nmol/L), which was true in males (16.11 ± 0.24 nmol/L vs. 16.85 ± 0.15 nmol/L; Figure 4B) but not in females (16.43 ± 0.38 nmol/L vs. 16.58 ± 0.11 nmol/L; Figure 4C). Figure 4D indicates that only anemic adults had significantly lower 25(OH)D_3_ than their non-anemic counterparts (15.81 ± 0.32 nmol/L vs. 17.41 ± 0.15 nmol/L). Again, this was true in males (15.26 ± 0.35 nmol/L vs. 17.55 ± 0.24 nmol/L; Figure 4E) but abolished in females (17.22 ± 0.71 nmol/L vs. 17.32 ± 0.20 nmol/L; Figure 4F).

### 3.5. Patterns of Serum Ca^2+^ in Anemic Subjects

**Figure 5:**
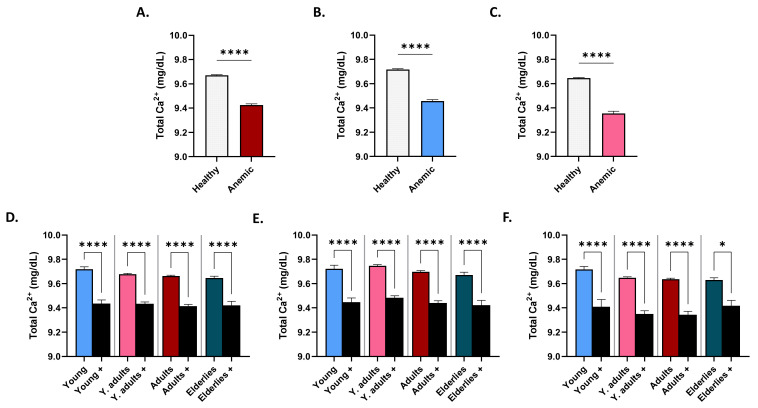
Ca^2+^ levels in healthy and anemic subjects. Mean ± SEM of serum Ca^2+^ in healthy and anemics (+) of both genders (**A**), of males (**B**), of females (**C**), in age groups of both genders (**D**), of males (**E**), and of females (**F**). * (*p* < 0.05) and **** (*p* < 0.0001) indicate significant difference, while ns indicates no significance.

Figure 5A shows that total serum Ca^2+^ was found to be significantly lower in anemic compared to non-anemic subjects (9.67 ± 0.01 mg/dL vs. 9.42 ± 0.01 mg/dL); a finding that was consistent when males (9.71 ± 0.01 mg/dL vs. 9.45 ± 0.01 mg/dL; Figure 5B) and females (9.64 ± 0.01 mg/dL vs. 9.35 ± 0.02 mg/dL; Figure 5C) were considered separately. Again, this pattern was observed across all age groups in both genders (Figure 5D), and when each gender was considered alone (Figure 5E,F).

### 3.6. Patterns of Serum Ferritin in Anemic Subjects

**Figure 6:**
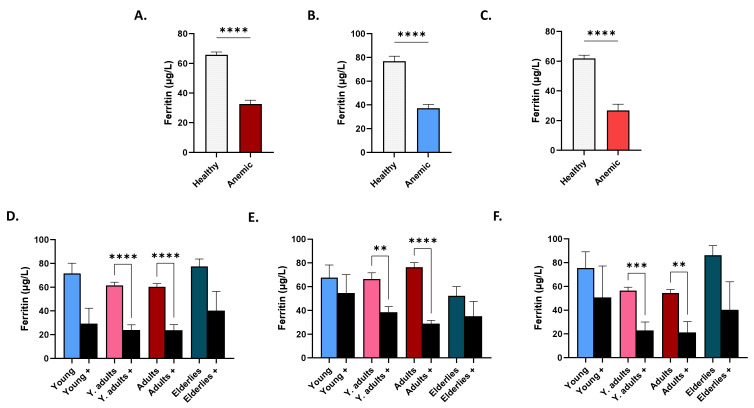
Ferritin levels in healthy and anemic subjects. Mean ± SEM of serum ferritin in healthy and anemics (+) of both genders (**A**), in males (**B**), in females (**C**), in age groups of both genders (**D**), of males (**E**), and of females (**F**). ** (*p* < 0.01), *** (*p* < 0.001), and **** (*p* < 0.0001) indicate significant difference, while ns indicates no significance.

Serum ferritin was significantly lower in anemic, as opposed to non-anemic, subjects (65.76 ± 1.94 μg/L vs. 32.66 ± 2.53 μg/dL), in both males (76.78 ± 4.20 μg/L vs. 37.29 ± 3.08 μg/L) and females (61.91 ± 2.16 μg/dL vs. 26.85 ± 4.18 μg/dL), as seen in Figure 6A–C. Age-wise comparisons revealed that, independent of gender, anemic young adults (61.48 ± 2.69 μg/L vs. 23.96 ± 4.31 μg/L) and adults (60.36 ± 2.62 μg/L vs. 23.74 ± 4.79 μg/L) had significant reductions in serum ferritin compared to non-anemic subjects of the same age group (Figure 6D). This was also true when either gender was considered alone (Figure 6E,F).

### 3.7. Patterns of Serum Iron in Anemic Subjects

**Figure 7:**
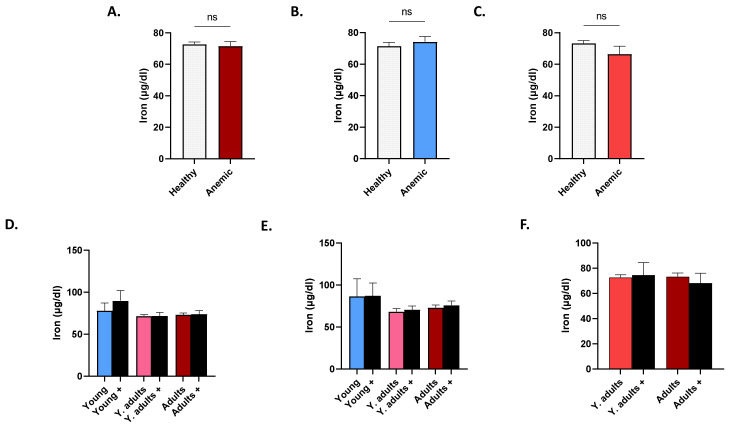
Iron levels in healthy and anemic subjects. Mean ± SEM of serum iron in healthy and anemics (+) of both genders (**A**), of males (**B**), of females (**C**), in age groups of both genders (**D**), of males (**E**), and of females (**F**). ns indicates no significance.

Figure 7 shows no significant changes in serum iron levels between anemic and non-anemic subjects, regardless of gender or age.

### 3.8. Prevalence of Serum 25(OH)D_3_, Ca^2+^, Ferritin, and Iron Disturbances in Anemia

As shown in Table 2, the prevalence of VDD in the studied population was extremely high, totaling 98.47%. Hypocalcemia was observed in 0.80% of all subjects, in 0.57% of non-anemics, and in 1.87% of anemics. Hypercalcemia was prevalent in 2.40% of all subjects, in 2.68% of non-anemics, and in 1.06% of anemics. Ferritin deficiency was detected in 51.11% of all subjects, in 40.32% of non-anemics, and in 90.70% of anemics. Iron deficiency prevalence was 36.36% in all subjects, 35.67% in non-anemics, and 40.74% in anemics.

### 3.9. Association of Serum 25(OH)D_3_, Ca^2+^, Ferritin, and Iron Disturbances in Anemia

**Figure 8:**
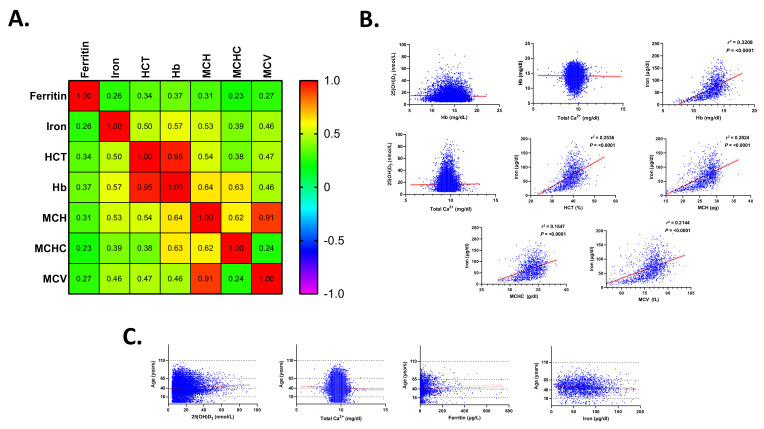
Correlation analysis of 25(OH)D_3_ and Ca^2+^ with anemia indices. (**A**) Pearson correlation matrix of ferritin and iron and CBC parameters. (**B**) Simple linear regression of 25(OH)D_3_, Ca^2+^, and iron with CBC parameters. (**C**) Simple linear regression of 25(OH)D_3_, Ca^2+^, ferritin, and iron with age.

As depicted in Figure 8A, Pearson correlation analysis revealed significant, positive association between ferritin and iron, hematocrit (HCT), Hb, mean corpuscular Hb (MCH), mean corpuscular Hb concentration (MCHC), and mean corpuscular volume (MCV). Iron was also significantly positively correlated with HCT, Hb, MCH, MCHC, and MCV. Furthermore, simple linear regression analysis (Figure 8B) indicated that, while no predictor of ferritin was found among the parameters analyzed, positive predictors of iron included Hb, HCT, MCH, MCHC, and MCV. No significant association was found among 25(OH)D_3_, Ca^2+^, ferritin, and iron (Figure 8B) irrespective of age (Figure 8C).

### 3.10. Risk Assessment of Serum 25(OH)D_3_, Total Ca^2+^, Ferritin, and Iron

Risk assessment of variables (Table 3) revealed that vitamin D-deficient subjects were 0.92 times more likely to be anemic and had 0.90 times the odds of being anemic than non-deficient subjects, although this association was not statistically significant (*p* > 0.05). The absolute risk of being anemic if hypocalcemic was 41.4%, and if normocalcemic was 17.5%. Hypocalcemic subjects were 2.36 times more likely to be anemic (i.e., hypocalcemics had a 97.7% chance of being anemic) and had 3.32 times the odds of being anemic than normocalcemic subjects. Conversely, there was statistical significance between hypercalcemia and anemia but this association was lost after correction for hematocrit. Furthermore, ferritin-deficient subjects were 9.33 times more likely to be anemic and had 14.44 times the odds of being anemic than their ferritin-sufficient counterparts.

## 4. Discussion

Serum 25(OH)D_3_ is widely considered the best estimate of body stores of vitamin D, as it reflects dietary and skin sources of vitamin D. We found widespread deficiency of vitamin D in the Saudi population, irrespective of age, gender, or anemia (Table 2). This remarkably high prevalence in the study population might have masked a potential role of the deficiency in discriminating anemics, although an association between vitamin D status and anemia would have been detected even in insufficiency or overt deficiency, which was not the case in the current study. Thus, a possible role of mediator variables, such as obesity, dyslipidemia, and erythropoietin, cannot be excluded [13]. Nevertheless, while still inadequate, vitamin D stores exhibited a statistically significant increasing trend with age (Figure 1B–D) in clear contrast to that shown by Ca^2+^ (Figure 1E–H). This was surprising as it has been shown that aging compromises 1,25[OH]_2_D renal synthesis, vitamin D receptor (VDR) activity and, subsequently, Ca^2+^ absorption [14]. One possible explanation was the increased multivitamin intake in the older age groups in different ethnicities [15], which might have contributed to the improved levels observed in this study compared to younger age groups. Studies on multivitamin consumption in the Saudi population are needed to confirm or rule out this possibility.

Although vitamin D may impact iron homeostasis, by suppressing inflammatory cytokines and hepcidin [16], the fact that PR and OR for anemia were 0.42 in hypercalcemia, despite the lack of statistical significance (Table 3), possibly indicated that the majority of the cases analyzed might have been unrelated to an underlying inflammatory process. This was supported by the finding that anemics had significantly diminished serum ferritin (Figure 6), but not iron (Figure 7); uncharacteristic of anemia of chronic disease. On the other hand, our study also showed that anemic male adults had significantly lower 25(OH)D_3_ levels than their non-anemic counterparts (Figure 4). Moreover, Ca^2+^ was consistently significantly reduced in anemics, regardless of age or gender (Figure 5), and increased levels were not associated with a lower risk for anemia after correction for hematocrit (Table 3), in contrast to previously reported findings [17]. The loss of statistical significance after correcting for hematocrit might be due to renal hemoconcentration, which falsely elevates Hb levels. Thus, a subset of patients may indeed have had anemia of chronic disease, in which VDD and its calciotropic effect may have had a significant role. It must be stressed, however, that it is unknown whether the anemia cases analyzed were corrected with supplementary iron with or without vitamin D, which precludes establishing conclusive evidence. Likewise, data on BMI, Ca^2+^ disorders, and the parathyroid status were unavailable, which is another important consideration. Renal impairment, hemoconcentration, inadequate Ca^2+^ intake, muscle wasting, hyperthyroidism, and low 25(OH)D_3_ trigger elevations in PTH to maintain Ca^2+^ levels [18]. In fact, secondary hyperparathyroidism and VDD are consequences of obesity [19,20], and if not coupled with an increase in PTH, diminished 25(OH)D_3_ levels may not reflect bona fide VDD [21]. In congruence, meta-analysis of randomized clinical trials has found that supplementation with vitamin D restores serum 25(OH)D_3_ and PTH to physiological levels [20] with higher doses required for obese subjects [22]. Likewise, obese adults taking cholecalciferol supplements exhibited a significant increase and decrease in serum 25(OH)D_3_ and PTH, respectively, but with no appreciable effect on Ca^2+^ or phosphate levels [23]. Furthermore, primary hyperparathyroidism patients showed improvement in PTH and bone resorption and mineral density following vitamin D supplementation [24]. Altogether, these findings underscore the relevance of adiposity and PTH and Ca^2+^ disorders to vitamin D homeostasis.

In a population study in Turkey, it was found that 25(OH)D_3_ levels were significantly lower in anemic subjects and in those with lower serum ferritin and iron [25]. VDD was also reported to be more prevalent in anemic female Egyptians compared to non-anemics, albeit with no significant correlation with iron status [26]. Similarly, Caucasian professional female athletes with VDD had significantly lower iron and ferritin levels than non-deficient participants [27]. In our study, we particularly found reduced 25(OH)D_3_ levels in anemics only when females were excluded (Figure 4B,E). Gender disparity in VDD was similarly observed in varying ethnicities, including obese Norwegian adult males [28], New Zealanders [29], and Brazilians [30]. Whether this variation could be, at least in part, attributed to ethnicity, dietary habits, physical activity, or solarium use, remains to be determined.

Although females had significantly lower serum iron level than males (Figure 2E), adult females were the only group that retained significance upon further age-wise comparison (Figure 2H). Increased demand for iron due to pregnancy might account for this observation, despite the possibility of being postmenopausal. Insufficient dietary intake, blood loss, and intrauterine device usage [31] are other possible causes. Of note, young adult and adult males and females had significantly lower ferritin levels compared to elderlies (Figure 2B,D); a pattern that persisted in anemics of both genders (Figure 6E,F). Taken together, it seems likely that this age group, in particular, are at an increased risk for depleted iron stores and that the cohort analyzed represented iron-deficiency anemia. We found that serum ferritin (Figure 6), but not iron (Figure 7), was able to discriminate anemics, which suggests correction with iron supplementation or early stages of iron-deficiency anemia. Both parameters, nonetheless, showed positive correlation with anemia indices (Figure 8A), but only changes in iron could be explained by those in anemia indices (Figure 8B). No association between 25(OH)D_3_ or Ca^2+^ and anemia parameters was found in our study, which was in agreement with a previous report showing that Ca^2+^ supplementation had no significant long-term effect on iron status [32]. Nonetheless, it seems plausible that the increased risk of anemia observed in hypocalcemics (Table 3) might be mediated through mechanisms unrelated to, or unexclusively reliant on, iron status, or that mediator and moderator variables may account for this observation, which remains to be explored in longitudinal studies.

The effect of body and fat mass on vitamin D and iron status cannot be overlooked as obesity is an established risk factor for VDD. Since vitamin D is fat-soluble, body stores are vulnerable to fluctuations in fat tissue [33] and vitamin D has several modulatory effects on adipose tissue, including gene expression, adipokine release, and adipocyte apoptosis [34]. In a meta-analytic review, the prevalence of VDD was 35% higher in obese, compared to lean, individuals. regardless of age [35]. A recent systematic review found a positive association between iron status and vitamin D, possibly through inhibition of calcitriol synthesis by hydroxylases [36]. Moreover, 25(OH)D_3_ and BMI were negatively associated in European males [37] and Chilean children [38], while muscle strength was positively associated with 25(OH)D_3_ in young, Irish males [39]. A significant, but weakly negative, correlation between BMI and 25(OH)D_3_ was also reported in adult populations [40]. This may be due to sequestration of the vitamin in adipose tissue which impairs release and bioavailability [41]. Notably, VDD also impacts obesity-associated insulin resistance and is an independent risk factor for hyperglycemia [42]. Possible mechanisms linking obesity to VDD include altered nutrient utilization, inflammatory damage leading to compromised iron absorption, due to increased hepcidin and ferritin, and plasma hypervolemia [21]. Since ferritin is elevated as a result of obesity-induced inflammation, underestimation of iron deficiency is not uncommon under this condition [43]. Despite this masking effect, iron deficiency has consistently been demonstrated to be more prevalent in overweight and obese children and adolescents as compared to lean subjects [43,44]. Moreover, iron malabsorption, as opposed to inadequate intake, may explain the lack of efficacy of iron supplementation to reverse iron deficiency in a number of clinical trials [36]. Thus, a comprehensive approach, combining iron markers, including serum iron, ferritin, total iron-binding capacity, and soluble transferrin receptor, is more appropriate to evaluate iron status in light of BMI and muscle and fat content.

The strengths of the study included the very large sample size, which was representative of the general population, and the automated data acquisition, which reduced analytical variability. The limitations of the study included the inability to determine causality, given the cross-sectional nature of the study, and the unavailability of multiple key variables, such as BMI, PTH, comorbidities and, most importantly, Ca^2+^ disorders, dietary intake, and medication use, which prevented adjustment for confounding except for age and gender. Longitudinal studies are, therefore, warranted to evaluate disturbances in vitamin D and Ca^2+^ as risk factors and determinants of anemia in the Saudi population.

In conclusion, this report demonstrates that VDD is alarmingly prevalent in the Saudi population and highlights a subsequent decreasing trend in Ca^2+^ levels with age. Serum 25(OH)D_3_ was significantly diminished only in anemic adult males, unlike Ca^2+^ which was consistently diminished in anemics of both genders and across all age groups. Hypocalcemia was found to be associated with an increased risk for anemia, and ferritin, but not iron, was depleted in young adult and adult anemics. Since exclusion of males displaced the significance of diminished 25(OH)D_3_ levels in anemics, clinicians must be aware of the effects of gender and age on 25(OH)D_3_ and Ca^2+^ within the context of anemia and guidelines that take into consideration these variables must be developed in light of emerging evidence from randomized controlled trials.

## Figures and Tables

**Table 1:** Age groups used in the study.

Age Group	Range (Years)	No. of Subjects
Males		
Young	0–17	435
Young adults *	18–39	2358
Adults	40–64	2599
Elderlies	65–110	563
Females		
Young	0–17	430
Young adults *	18–39	4320
Adults	40–64	2870
Elderlies	65–110	629

* A total of 25 young adults were of unknown gender.

**Table 2:** Prevalence of 25(OH)D_3_, Ca^2+^, ferritin, and iron disturbances.

Condition	Prevalence (%)		
	All Subjects	Healthy	Anemic
VDD	98.47	98.49	98.34
Hypocalcemia	0.80	0.57	1.87
Hypercalcemia	2.40	2.68	1.06
Ferritin deficiency	51.11	40.32	90.70
Iron deficiency	36.36	35.67	40.74

**Table 3:** Association of disturbed 25(OH)D_3_, Ca^2+^, ferritin, and iron with anemia.

Condition	PR	95% CI	*p*	OR	95% CI	*p*
VDD	0.92	0.69–1.22	0.5749	0.90	0.64–1.27	0.5782
Hypocalcemia	2.36	1.88–2.95	<0.0001	3.32	2.27–4.86	<0.0001
Hypercalcemia	0.42	0.10–1.70	0.2257	0.42	0.10–1.70	0.2240
Ferritin deficiency	9.33	6.27–13.87	<0.0001	14.44	9.38–22.22	<0.0001
Iron deficiency	1.20	0.59–2.44	0.6102	1.24	0.54–2.84	0.6114

## Data Availability

Data is available from the corresponding author upon reasonable request, and with permission of Al-Borg Medical Laboratories.
